# Detection of Inferred CCR5- and CXCR4-Using HIV-1 Variants and Evolutionary Intermediates Using Ultra-Deep Pyrosequencing

**DOI:** 10.1371/journal.ppat.1002106

**Published:** 2011-06-23

**Authors:** Evelien M. Bunnik, Luke C. Swenson, Diana Edo-Matas, Wei Huang, Winnie Dong, Arne Frantzell, Christos J. Petropoulos, Eoin Coakley, Hanneke Schuitemaker, P. Richard Harrigan, Angélique B. van 't Wout

**Affiliations:** 1 Department of Experimental Immunology, Sanquin Research, Landsteiner Laboratory, Center for Infection and Immunity Amsterdam, Academic Medical Center, University of Amsterdam, Amsterdam, The Netherlands; 2 British Columbia Centre for Excellence in HIV/AIDS, Vancouver, British Columbia, Canada; 3 Monogram Biosciences, South San Francisco, California, United States of America; University of Zurich, Switzerland

## Abstract

The emergence of CXCR4-using human immunodeficiency virus type 1 (HIV-1) variants is associated with accelerated disease progression. CXCR4-using variants are believed to evolve from CCR5-using variants, but due to the extremely low frequency at which transitional intermediate variants are often present, the kinetics and mutational pathways involved in this process have been difficult to study and are therefore poorly understood. Here, we used ultra-deep sequencing of the V3 loop of the viral envelope in combination with the V3-based coreceptor prediction tools PSSM_NSI/SI_ and geno2pheno_[coreceptor]_ to detect HIV-1 variants during the transition from CCR5- to CXCR4-usage. We analyzed PBMC and serum samples obtained from eight HIV-1-infected individuals at three-month intervals up to one year prior to the first phenotypic detection of CXCR4-using variants in the MT-2 assay. Between 3,482 and 10,521 reads were generated from each sample. In all individuals, V3 sequences of predicted CXCR4-using HIV-1 were detected at least three months prior to phenotypic detection of CXCR4-using variants in the MT-2 assay. Subsequent analysis of the genetic relationships of these V3 sequences using minimum spanning trees revealed that the transition in coreceptor usage followed a stepwise mutational pathway involving sequential intermediate variants, which were generally present at relatively low frequencies compared to the major predicted CCR5- and CXCR4-using variants. In addition, we observed differences between individuals with respect to the number of predicted CXCR4-using variants, the diversity among major predicted CCR5-using variants, and the presence or absence of intermediate variants with discordant phenotype predictions. These results provide the first detailed description of the mutational pathways in V3 during the transition from CCR5- to CXCR4-usage in natural HIV-1 infection.

## Author Summary

The first step in the infection of a target cell by human immunodeficiency virus type 1 (HIV-1) is binding of the envelope spike to its receptor CD4 and a coreceptor on the cellular surface. HIV-1 variants present early in the course of infection mainly use the coreceptor CCR5, while virus variants that use CXCR4 can appear later in infection. This change in coreceptor usage is associated with mutations in the third variable (V3) loop of the envelope spike, but has been difficult to study due to the low presence of intermediate variants. Using ultra-deep sequencing, we obtained thousands of sequences of the V3 loop from HIV-1 infected individuals in the year before CXCR4-using variants were first detected, including sequences from almost all intermediate variants. We show that mutations are introduced sequentially in the V3 loop during the evolution from CCR5- to CXCR4-usage. Furthermore, we describe differences and similarities between HIV-1-infected individuals that are related to this change in coreceptor usage, which provides the first detailed overview of this evolutionary process during natural HIV-1 infection.

## Introduction

The entry of human immunodeficiency virus type 1 (HIV-1) into a target cell is dependent on the binding of the envelope glycoprotein to its receptor CD4 and a coreceptor, either CCR5 or CXCR4. Although the reasons are incompletely understood, primary HIV-1 infection is predominantly established by CCR5-using (R5) HIV-1 variants [1]–[4]. In approximately half of the individuals infected with subtype B HIV-1, CXCR4-using (X4) variants evolve from R5 viruses during the asymptomatic phase of infection, and their emergence coincides with an accelerated progression to AIDS [5]–[8]. This evolution from CCR5-usage to CXCR4-usage often goes through intermediate variants that are able to use both coreceptors. These R5X4 viruses can be further classified according to the efficiency of their coreceptor usage as Dual-R (more efficient use of CCR5) or Dual-X (more efficient use of CXCR4) [9]. Pure R5 variants remain present after the appearance of CXCR4-using variants, and in the vast majority of HIV-infected individuals both virus populations co-exist during the remaining course of infection [10], [11].

Despite years of research, the mechanisms involved in the appearance of CXCR4-using viruses remain to be fully understood. The main determinants for coreceptor usage are located in the second (V2) and third (V3) variable loop of Env [12]–[16], but changes in C2 [17], [18], C4 [19] and even in gp41 [17], [20] have also been reported to influence coreceptor usage. In particular, positively charged amino acid residues at positions 11 and/or 25 of the V3 loop are highly associated with CXCR4-usage [21], [22]. Although as few as one or two amino acid substitutions may be sufficient to change coreceptor usage [22]–[24], the earliest detectable CXCR4-using viruses *in vivo* show evidence of additional, compensatory, mutations [25]. Together with a decreased replication rate and reduced coreceptor efficiency of intermediate variants [17], [25], [26], these findings suggest that the transition from CCR5- to CXCR4-usage involves a phase of markedly reduced viral fitness.

The presence or absence of CXCR4-using virus populations in infected individuals can be monitored using phenotype-based methods, such as the PBMC-based MT-2 assay [27], [28] and the plasma-based recombinant Trofile assay [29], [30]. In addition, genotype-based detection methods using signature changes in the sequence of the V3 loop of CXCR4-using variants [21], [22], [31], [32] have been developed [33], [34]. However, transitional intermediate variants, which may be present at extremely low levels due to their low replication capacity, are likely to be overlooked by standard phenotype-based or genotype-based detection methods, which has precluded their characterization and has hampered our understanding of the transition from CCR5- to CXCR4-usage.

As deep sequencing technologies can provide multiple orders of magnitude greater coverage than conventional sequencing, we used this technique in combination with V3-based coreceptor prediction tools to detect HIV-1 variants during the transition from CCR5- to CXCR4-usage. We previously carefully characterized the first detection of CXCR4-using virus in ten HIV-1-infected individuals using the MT-2 assay and the original and enhanced-sensitivity Trofile assays on longitudinal PBMC and serum samples [35]. Here, we analyzed PBMC and serum samples obtained from the same group of subjects at three-month intervals up to one year prior to the first phenotypic detection of CXCR4-using variants in the MT-2 assay. The availability of thousands of clonal sequences per sample obtained at relatively short intervals allowed us to study the kinetics and mutational pathways involved in the emergence of CXCR4-using variants.

## Results

### Comparison between Genotypic Prediction of HIV-1 Coreceptor Usage and *In Vitro* Coreceptor Phenotype Determination

To determine whether we could use a V3-based prediction of coreceptor usage to detect CXCR4-using variants by deep sequencing on this set of HIV-infected individuals, we first validated our prediction tools using V3 sequences with a known coreceptor phenotype. To this end, recombinant viruses were generated from 21–63 clonal *env* sequences that were isolated from sera obtained from nine of our subjects (all individuals except DS6) at several time points before, at, and after the moment at which the MT-2 assay for the first time indicated the presence of CXCR4-using variants (time point zero). These virus clones were subsequently tested for their coreceptor usage in the Trofile assay (Monogram Biosciences). All individuals harbored both R5 and Dual-X variants at the later time points (**Table S1 – S9**). In addition, the emergence of Dual-X variants was preceded by Dual-R variants in five out of nine subjects (**Table S1 – S9**).

The coreceptor usage of the corresponding V3 sequences was subsequently inferred using two different bioinformatic tools: position-specific scoring matrix (PSSM_NSI/SI_) [33] and geno2pheno_[coreceptor]_ (g2p) [34]. For all individuals except DS9 and DS10, the phenotypes of (nearly) all R5 and Dual-X Env variants were predicted correctly by both tools (i.e. nsi/r5 or si/x4, respectively; **Table 1** and **Table S1 – S9**). The three exceptions are one R5 variant and two Dual-R variants with predicted si/x4 phenotypes in subject DS3 and subject DS8, respectively. On the other hand, the V3 sequences of Dual-R Env variants were in general identical to those of co-existing R5 variants, and were consequently predicted to have an nsi/r5 phenotype. In addition, a subset of R5 and Dual-R variants with identical V3 sequences from subject DS8 had an si/r5 phenotype. Dual-R viruses showed much lower infectivity on the CXCR4 cell line than on the CCR5 cell line, and previous work has demonstrated that the determinants of coreceptor usage in these viruses are most likely located outside of the V3 region [9]. Because they could not be distinguished from R5 viruses on the basis of V3 sequence, they were categorized as CCR5-using variants for the purposes of this analysis.

**Table 1:** Predicted phenotypes of Env clones for which coreceptor usage was determined in the Trofile assay.

ACS ID	Subject	*n* clones	Phenotype Trofile	Predicted phenotype (PSSM/g2p)^a^
H13912	DS1	15	R5	nsi/r5
		6	Dual-X	si/x4
H13988	DS2	49	R5	nsi/r5
		3	Dual-X	si/x4
H13845	DS3	26	R5	nsi/r5
		1	R5	si/x4
		20	Dual-R	nsi/r5
		1	Dual-R	si/r5
		15	Dual-X	si/x4
H13951	DS4	36	R5	nsi/r5
		6	Dual-R	nsi/r5
		12	Dual-X	si/x4
H13993	DS5	42	R5	nsi/r5
		9	Dual-X	si/x4
H13885	DS6	n.a.	n.t.	n.t.
H13907	DS7	19	R5	nsi/r5
		4	Dual-X	si/x4
H13908	DS8	17	R5	nsi/r5
		3	R5	si/r5
		2	Dual-R	nsi/r5
		8	Dual-R	si/r5
		2	Dual-R	si/x4
		1	Dual-X	si/r5
		5	Dual-X	si/x4
H13904	DS9	46	R5	nsi/x4
		3	R5	nsi/r5
		1	Dual-R	nsi/x4
		10	Dual-X	nsi/x4
H13940	DS10	6	R5	nsi/r5
		9	R5	si/r5
		11	R5	si/x4
		17	Dual-R	si/x4
		1	Dual-R	si/r5
		5	Dual-X	si/x4

a Cutoffs for PSSM and g2p were −1.75 and 3.5%, respectively.

ACS ID, subject identifier of Amsterdam Cohort Studies; *n*, number; n.a., not applicable; n.t., not tested.

In subject DS9, 57 of 60 (95%) virus variants were called nsi by PSSM and x4 by g2p, irrespective of their *in vitro* phenotype (**Table 1** and **Table S8**), while the coreceptor usage of only three clones was predicted correctly (nsi by PSSM and r5 by g2p). For subject DS10, a large proportion of R5 viruses (42%) were incorrectly predicted to be CXCR4-using by both PSSM and g2p (**Table 1** and **Table S9**). Assuming that the Trofile assay reported the correct phenotype, the prediction tools could not distinguish between phenotypically distinct variants in DS9 and DS10, and these two individuals were therefore excluded from further analysis.

### Detection of Predicted CXCR4-Using V3 Sequences by Ultra-Deep Sequencing

For the eight remaining subjects, V3 sequences were generated from PBMC and serum samples obtained at three-monthly intervals between 12 months before and time point zero by 454-sequencing. Per sample, a median of 7,123 reads with a frequency of ≥3 were obtained (range, 3,482–10,521; **Tables 2** and **3**). The majority (median 70%; range, 33–100%) of sequences detected at one time point with a frequency >10% in one compartment (PBMC or serum) were also detected at the subsequent time point in the same compartment (**Figure S1**). In general, the percentage of sequences that were detected at two consecutive time points was lower for PBMC samples than for serum samples (**Figure S1**), which may reflect a lower input number of HIV copies in PBMCs and is indicative of a larger sampling bias for PBMC samples. In addition, in subjects DS6 and DS8 (who had relatively low viral loads) and DS2 (for whom viral load measurements were not available), the percentage of sequences in serum that was detected at two consecutive time points was lower as compared to the remaining individuals. This was observed in particular for the sequences that were present at low frequencies (<1%), although some sequences that were not detected in serum were present in PBMC at the next time point.

**Table 2:** CD4^+^ T cell counts, viral load and MT-2 assay results of participants DS1–DS4 around and during the period of study.

Subject	Time to SI (mo)	CD4+ T cells/µl blood	RNA load/ml plasma	MT-2	Reads PBMC	Reads serum
DS1	−20.9	530	26,000	n.t.	n.a.	n.a.
	−18.0	590	n.t.	NSI	n.a.	n.a.
	−14.5	800	n.t.	NSI	n.a.	n.a.
	−11.7	520	n.t.	NSI	n.t.	5,754
	−8.3	450	42,000	NSI	3,660	8,659
	−6.0	390	n.t.	NSI	n.t.	3,482
	−3.0	410	n.t.	NSI	5,383	3,497
	0.0	570	n.t.	SI	5,472	5,126
	3.2	360	99,000	SI	n.a.	n.a.
	6.1	300	n.t.	SI	n.a.	n.a.
	9.1	220	n.t.	SI	n.a.	n.a.
DS2	−19.3	480	n.t.	NSI	n.a.	n.a.
	−15.2	340	n.t.	NSI	n.a.	n.a.
	−12.2	460	n.t.	NSI	9,840	6,589
	−8.9	300	n.t.	NSI	8,098	8,135
	−5.8	440	n.t.	NSI	8,570	5,138
	−2.8	580	n.t.	NSI	6,932	5,489
	0.0	480	n.t.	SI	10,076	6,269
	3.0	440	n.t.	SI	n.a.	n.a.
	6.0	280	n.t.	SI	n.a.	n.a.
	8.9	320	n.t.	SI	n.a.	n.a.
DS3	−9.2	800	23,000	NSI	6,064	5,731
	−6.0	990	n.t.	NSI	9,027	6,482
	−3.0	1,230	120,000	NSI	9,364	7,448
	0.0	890	100,000	SI	10,316	7,947
	3.0	910	530,000	SI	n.a.	n.a.
	6.0	650	1,300,000	SI	n.a.	n.a.
	9.0	650	860,000	SI	n.a.	n.a.
DS4	−21.1	710	n.t.	fail	n.a.	n.a.
	−18.1	680	n.t.	NSI	n.a.	n.a.
	−15.1	560	n.t.	NSI	n.a.	n.a.
	−11.9	790	76,000	NSI	6,223	5,371
	−8.9	590	n.t.	NSI	n.t.	6,346
	−6.2	490	n.t.	NSI	9,507	8,654
	−2.8	530	n.t.	NSI	10,267	9,247
	0.0	330	n.t.	SI	10,521	6,550
	3.0	400	n.t.	SI	n.a.	n.a.
	6.0	370	120,000	SI	n.a.	n.a.
	9.3	230	n.t.	SI	n.a.	n.a.

SI, syncytium-inducing; NSI, non-syncytium-inducing; mo, months; n.t., not tested; n.a., not applicable.

**Table 3:** CD4^+^ T cell counts, viral load and MT-2 assay results of participants DS5–DS8 around and during the period of study.

Subject	Time to SI (mo)	CD4+ T cells/µl blood	RNA load/ml plasma	MT-2	Reads PBMC	Reads serum
DS5	−20.9	660	n.t.	NSI	n.a.	n.a.
	−17.9	790	14,000	NSI	n.a.	n.a.
	−14.9	600	5,900	NSI	n.a.	n.a.
	−12.0	490	2,400	fail	10,239	8,881
	−9.0	530	6,100	NSI	8,932	8,734
	−6.0	450	6,100	NSI	9,623	9,585
	−3.0	410	7,600	NSI	8,906	9,083
	0.0	520	17,000	SI	7,556	8,662
	2.8	450	6,700	SI	n.a.	n.a.
	5.6	480	80	NSI	n.a.	n.a.
	8.8	580	3,600	NSI	n.a.	n.a.
DS6	−18.5	660	n.t.	NSI	n.a.	n.a.
	−15.3	440	n.t.	NSI	n.a.	n.a.
	−12.1	330	n.t.	NSI	6,959	7,671
	−9.1	310	2,100	NSI	7,500	7,772
	−6.1	460	n.t.	NSI	7,287	6,320
	−3.2	470	n.t.	NSI	7,645	5,945
	0.0	470	n.t.	SI	6,930	6,179
	3.0	470	n.t.	SI	n.a.	n.a.
	6.0	310	10,000	SI	n.a.	n.a.
	9.0	280	23,000	SI	n.a.	n.a.
DS7	−19.9	780	n.t.	n.t.	n.a.	n.a.
	−16.7	670	n.t.	n.t.	n.a.	n.a.
	−13.7	490	n.t.	n.t.	n.a.	n.a.
	−10.9	640	n.t.	NSI	5,049	6,909
	−8.1	740	n.t.	NSI	4,765	3,489
	−5.2	500	n.t.	NSI	n.t.	5,045
	−2.8	670	n.t.	NSI	3,818	4,953
	0.0	630	120,000	SI	6,901	5,139
	3.2	510	n.t.	SI	n.a.	n.a.
	6.1	430	n.t.	SI	n.a.	n.a.
	9.1	380	n.t.	SI	n.a.	n.a.
	12.3	130	340,000	SI	n.a.	n.a.
DS8	−19.9	520	n.t.	n.t.	n.a.	n.a.
	−16.7	330	n.t.	n.t.	n.a.	n.a.
	−12.5	420	n.t.	NSI	9,901	6,666
	−10.7	390	n.t.	NSI	6,184	6,835
	−7.1	430	n.t.	NSI	9,706	7,641
	−3.0	400	n.t.	NSI	10,169	8,292
	0.0	270	n.t.	SI	9,474	5,522
	3.0	240	1,000	SI	n.a.	n.a.
	4.1	320	n.t.	SI	n.a.	n.a.
	6.8	330	1,000	SI	n.a.	n.a.
	9.7	190	5,800	SI	n.a.	n.a.

SI, syncytium-inducing; NSI, non-syncytium-inducing; mo, months; n.t., not tested; n.a., not applicable.

The coreceptor use of V3 sequences obtained by ultra-deep sequencing was subsequently inferred by PSSM and g2p. A high degree of concordance was observed between the two algorithm predictions among the V3 sequences of five subjects: DS1, DS2, DS4, DS6, and DS7. In contrast, relatively large discrepancies (≥15% of all reads per sample) between the predictions by PSSM and g2p (i.e. nsi/x4 or si/r5) were observed for at least one sample in subjects DS3, DS5, and DS8. To prevent an overestimation of the percentage CXCR4-using HIV-1 variants, V3 sequences were conservatively defined to be CXCR4-using when PSSM and g2p were concordant in si/x4 prediction.

**Figure 1:**
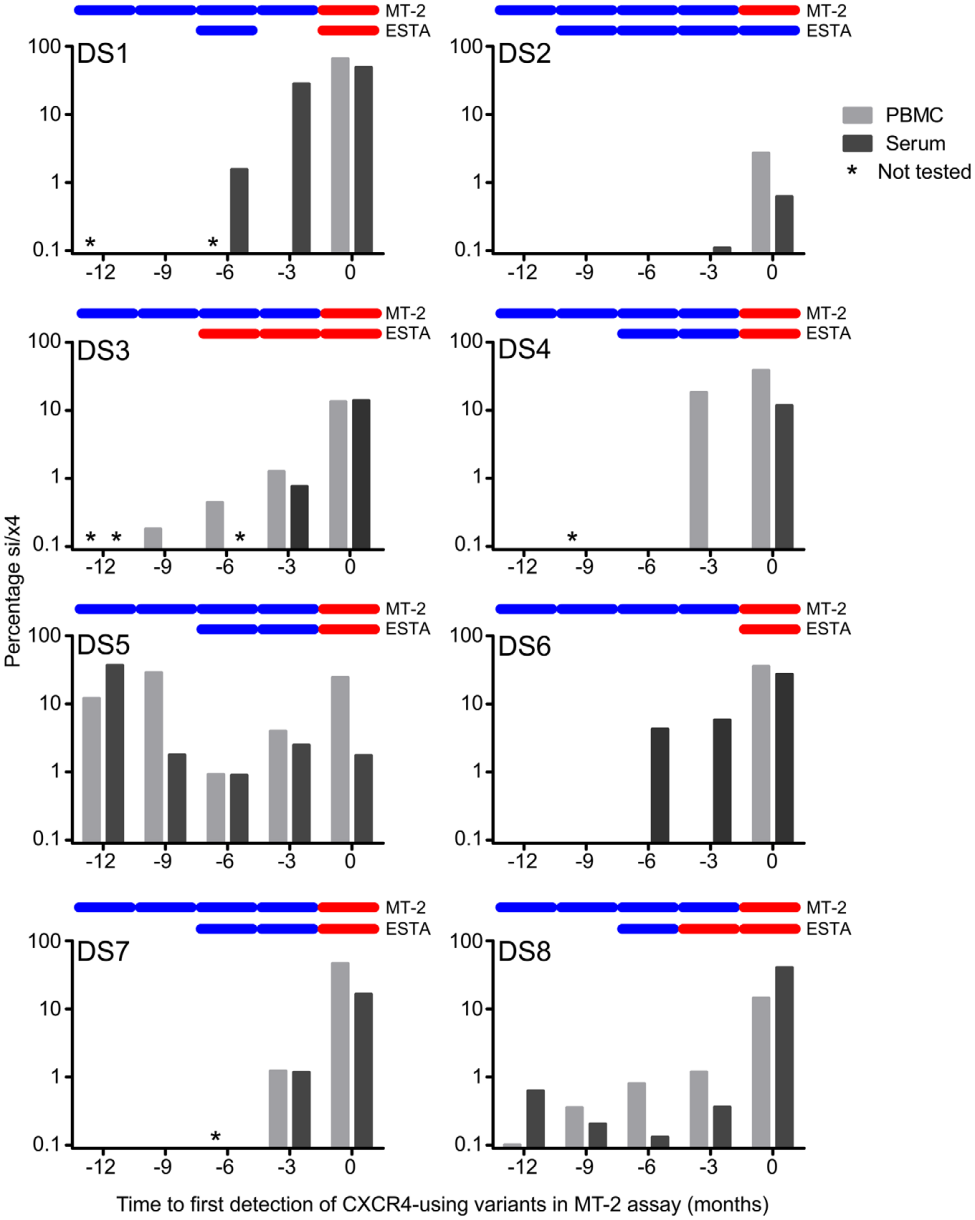
Detection of predicted CXCR4-using variants that emerge during natural HIV-1 infection using deep sequencing. For each participant, the percentage si/x4 sequences in PBMC (light grey bars) and serum (dark grey bars) at time points up to one year before the first phenotypic detection of CXCR4-using variants in the MT-2 assay are shown. Samples that have not been sequenced are indicated with an asterisk. In the top of each graph, the results of the MT-2 assay and the enhanced-sensitivity Trofile assay (ESTA) are given: a blue bar indicates NSI or R5, respectively, while a red bar indicates SI or D/M, respectively.

In these eight individuals, si/x4 sequences were detected at least three months prior to phenotypic detection in the MT-2 assay and were found as early as 12 months before time point zero in two of eight subjects (**Figure 1**). In three individuals (DS1, DS2, and DS6), these si/x4 variants were not detectable in PBMCs, which has most likely precluded their detection in the PBMC-based MT-2 assay. In total, in 13 of 27 PBMC samples obtained before time point zero we detected the presence of si/x4 variants at levels between 0.10% and 29% (median 1.2%). The inability of the MT-2 assay to detect high levels of si/x4 variants may be a result of low replication rates of these virus variants on the MT-2 cell line. In addition, si/x4 variants were observed in 16 of 30 serum samples obtained before time point zero. Of the serum samples obtained before time point zero that were analyzed in the enhanced-sensitivity Trofile assay (ESTA) and that were used for ultra-deep sequencing (*n* = 13), seven samples showed concordant results with our genotypic data, while six samples that previously scored R5 were shown to contain si/x4 variants at levels between 0.13% and 2.5% (**Figure 1**). The sensitivity of the ESTA varied between subjects, for example giving a positive result for the −3 months serum sample from DS8 (0.4% si/x4 sequences) but a negative result for the −3 month serum sample from DS5 (2.5% si/x4 sequences). This variation in detection limit is most likely the result of differences between infectivity of viral envelopes from different individuals on the U87 indicator cell lines.

### Timing of Detection of Predicted CXCR4-Using V3 Sequences in Serum and PBMC

**Figure 2:**
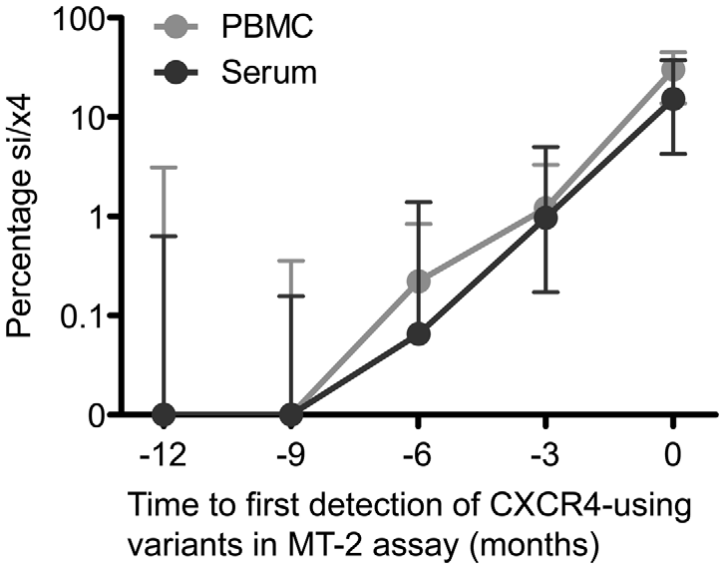
Increasing percentage of predicted CXCR4-using variants over time following their appearance during natural HIV-1 infection. The median percentage si/x4 sequences generated from PBMC (light grey) and serum (dark grey) by deep sequencing at time points up to one year before the first phenotypic detection of CXCR4-using variants in the MT-2 assay in eight participants are shown. Error bars represent the interquartile ranges.

In four of eight individuals, V3 sequences with an si/x4 phenotype emerged in both serum and PBMC at the same time point (**Figure 1**). In DS1 and DS6, the first si/x4 sequences were detected in serum six months prior to the appearance of si/x4 sequences in PBMC at levels between 1.6 and 4.3%, while a very small percentage of si/x4 sequences (0.1%) appeared in serum three months before their detection in PBMC in subject DS2. In contrast, si/x4 sequences (18.3% of the total number of reads) were observed in PBMC three months earlier than in serum in subject DS4. In general, the percentage of si/x4 sequences in both serum and PBMC increased over time (**Figure 2**). In agreement with previous findings [36], [37], six of eight individuals showed a higher prevalence of si/x4 sequences in PBMC (range, 2.7–66.0%) than in serum (range, 0.6–49.1%) at time point zero, while si/x4 sequences were more abundant in serum (range, 14.0–40.6%) than in PBMC (range, 13.4–14.5%) in the remaining two individuals.

### Evolution of nsi/r5 Sequences to si/x4 Sequences

From each sample, we obtained several hundreds of unique V3 reads. We first constructed neighbor-joining (NJ) trees using all unique reads obtained from the different time points and compartments per individual to exclude contamination of samples (data not shown). However, these trees were too large and too complex to study the genetic relationship of our sequences, and did not convey a good representation of the relative abundance of each V3 sequence. Therefore, we subsequently constructed minimum spanning trees (MSTs). MSTs are connection-type networks which are based on a model explaining sequence evolution in as few events as possible, similar to maximum parsimony (MP) algorithms [38], [39]. A MST thus represents the shortest possible combination of nucleotide changes between all sequences in the alignment. In contrast to most other methods for inferring evolutionary relationships, such as NJ or MP, MSTs do not contain hypothetical internal nodes. This type of analysis therefore requires all intermediate samples to be present in the total pool of sequences. As a result, MSTs can only be used for the analysis of sequences that show a limited degree of evolution and that are sampled frequently enough, and are less suitable for the analysis of, for example, full-length gp160 sequences in which a multitude of nucleotide substitutions as well as large insertions and deletions are observed over time. Due to these restrictions, MSTs turned out to be a powerful tool to visualize the genetic relationships between our closely related nsi/r5 sequences and si/x4 sequences of the V3 loop (comprising 105 nucleotides) and to identify intermediate sequence variants. Indeed, in the majority of individuals, all intermediate variants between the major nsi/r5 variant and the major si/x4 variant were found, while a maximum of two intermediate variants remained undetected in the other individuals.

**Figure 3:**
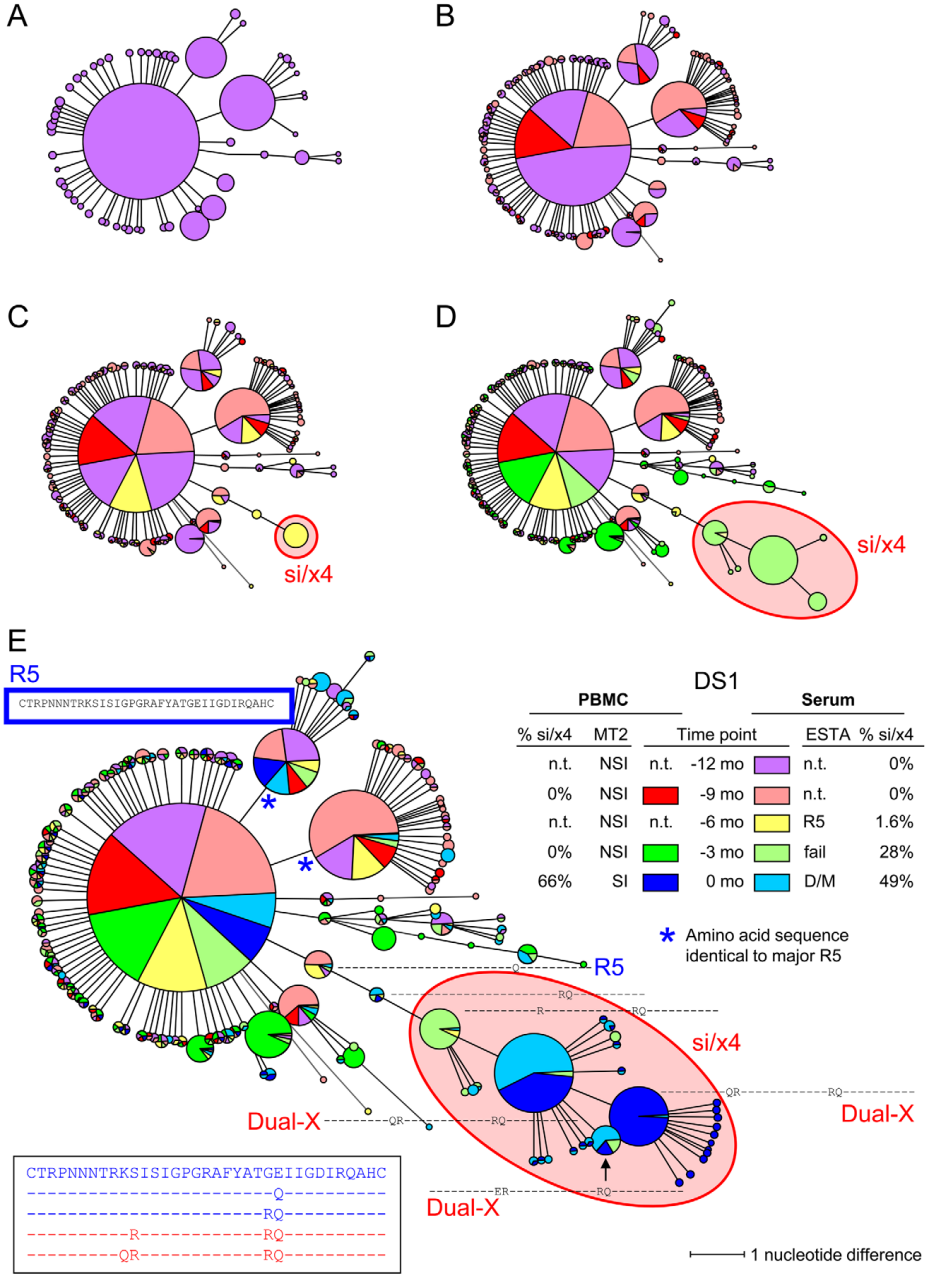
Minimum spanning tree (MST) of V3 sequences from subject DS1. V3 sequences generated by deep sequencing were used to construct MSTs. Identical nucleotide sequences are grouped in one node, and the circle size is proportional to the abundance of that particular V3 sequence. The length of the connecting branches corresponds to the number of nucleotide differences between the two connected nodes. Time points are color-coded, using bright colors for PBMC samples and corresponding soft colors for serum samples. (A) MST stripped of sequences from all time points except time point −12 months. For this time point, PBMCs were not available, and the MST thus consists of V3 sequences generated from serum only (shown in light red). (B) V3 sequences from PBMCs (shown in bright orange) and serum (shown in light orange) obtained at time point −9 months were added to the MST presented in panel A. (C) MST containing V3 sequences generated from −6 months serum (shown in light yellow) in addition to the sequences present in the MST in panel B. (D) Sequences from PBMCs (bright green) and serum (light green) at timepoint −3 months have been added to the MST in panel C. (E) Complete MST containing PBMC and serum V3 sequences from all time points. V3 amino acid sequences are shown relative to the majority sequence in PBMCs at the first time point (shown in blue box). Sequences of the major nsi/r5 variant, the major si/x4 variant, and all intermediates are additionally shown in the bottom left. In all panels, nodes containing V3 sequences with an si phenotype as inferred by PSSM_NSI/SI_ and an x4 phenotype as inferred by geno2pheno_[coreceptor]_ are indicated in red, all other sequences were predicted to be nsi/r5. The phenotype of V3 sequences of which the corresponding Env clone was analyzed in the Trofile assay is given in bright blue (for R5 sequences) or in bright red (for Dual-X sequences). Also shown are the results of phenotyping of PBMC samples by the MT-2 assay (NSI or SI) and of serum samples by ESTA (R5 or D/M), and the percentages si/x4 sequences obtained from PBMC and serum samples by deep sequencing (panel E, right side). N.t., not tested; mo, months.

For each subject, one MST was constructed including V3 nucleotide sequences generated from all time points of both serum and PBMC samples. A step-by-step explanation on how we read and interpret these MSTs is presented in **Figure 3** for subject DS1, and a summary of our observations is shown in **Table 4**. In subject DS1, one nsi/r5 sequence dominated at all time points in both serum and PBMCs, representing at least 30% (and up to 89%) of all sequences per time point. In addition, a population of closely related si/x4 variants was observed, of which the first variant appeared in serum at six months prior to the first positive MT-2 time point. At the later time points, this variant was still only detected in serum, while si/x4 variants with additional mutations appeared in serum at the next time point and in PBMCs at time point zero. Interestingly, the virus in subject DS1 required only three mutations in V3 to change from the existing nsi/r5 phenotype at −12 months to an si/x4 phenotype, which were introduced sequentially. After the introduction of the third of these substitutions, replacing the serine residue at position 11 of the V3 loop by an arginine residue, the PSSM and g2p predictions simultaneously switched from a CCR5-using inferred phenotype to a CXCR4-using inferred phenotype.

**Table 4:** Summary of the evolution of nsi/r5 sequences to si/x4 sequences obtained by ultra-deep sequencing.

Subject	Time pointfirst si/x4variant	Compartmentfirst si/x4variant	*n* major si/x4branchesin MST	*n* majornsi/r5variants^a^	Intermediatensi/x4 orsi/r5 variants	Minorsi/x4variants
DS1	−6	serum	1	3	no	no
DS2	−3	serum	1	11	yes	yes
DS3	−9	PBMC+serum	2	21	yes	yes
DS4	−3	PBMC	1	8	yes	no
DS5	−12	PBMC+serum	1	7	yes	no
DS6	−6	serum	2	14	yes	no
DS7	−3	PBMC+serum	1	11	no	no
DS8	−12	PBMC+serum	1	19	yes	yes

a The total number of major nsi/r5 variants that represents more than 80% of all nsi/r5 reads for each compartment at each time point. It is important to note that this number of major nsi/r5 variants does not always correspond to the number of (relatively) large nodes in the MSTs. The nodes in the MSTs are often made up of sequences from multiple time points and/or compartments, and the size of the node therefore does not always represent the frequency of the V3 sequence in an individual sample.

### Diversity in Major nsi/r5 Variants

**Figure 4:**
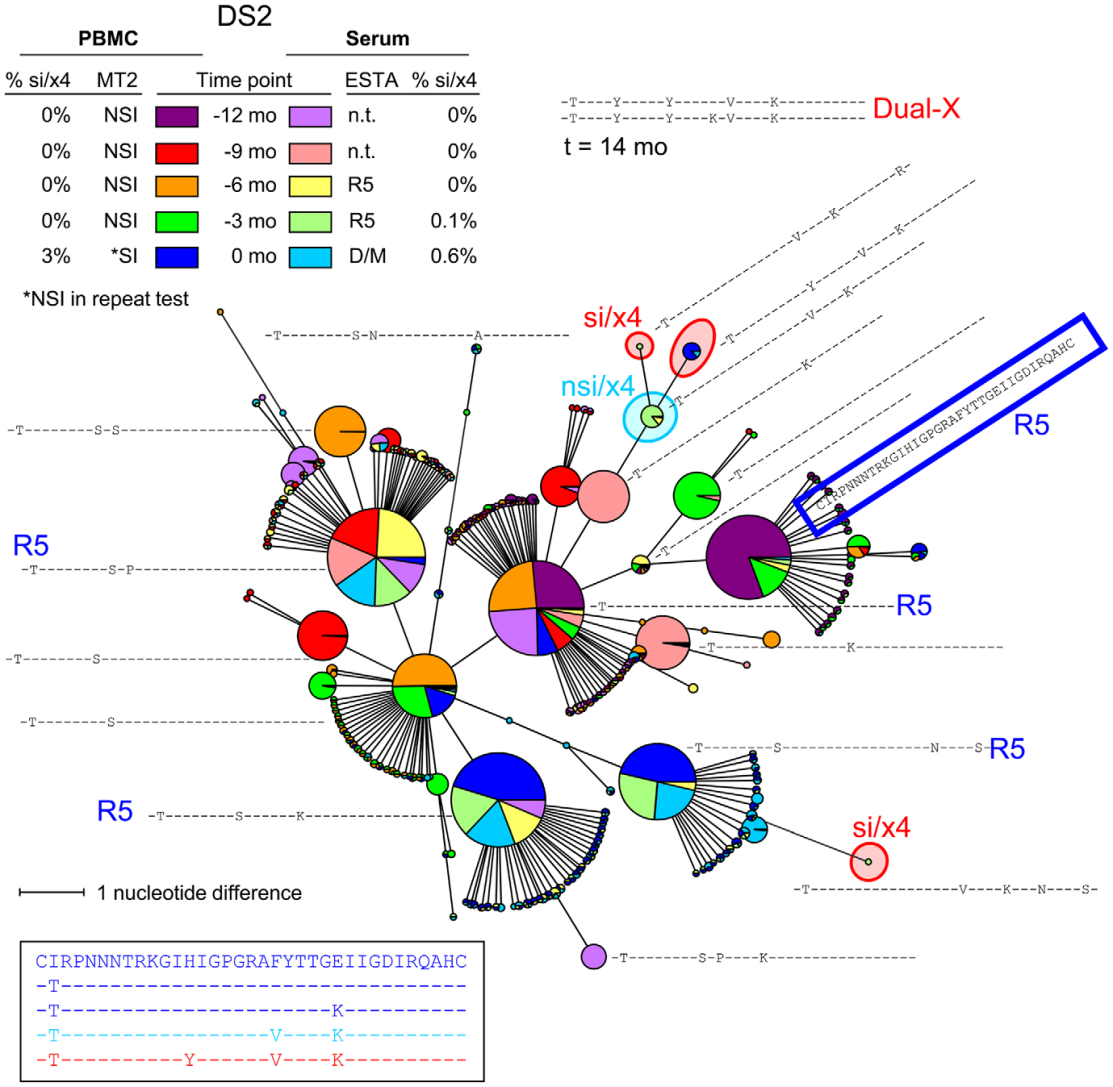
Relatively large variation among major nsi/r5 variants over time in subject DS2. An MST of all V3 sequences generated from PBMC and serum samples of all time points is shown. See the legend to Figure 3 for details regarding the layout of the figure. Nodes containing V3 sequences with an si phenotype as inferred by PSSM_NSI/SI_ and an x4 phenotype as inferred by geno2pheno_[coreceptor]_ are indicated in red, nodes containing discordant nsi/x4 variants are indicated in light blue, while all other sequences were predicted to be nsi/r5. As Dual-X clones were not detected in the Trofile assay until 18 months after the first MT-2^+^ time point, the V3 sequences of two Dual-X clones from this later time point are indicated in the top. These Dual-X sequences are closely related to the si/x4 sequences present at time point zero, suggesting that these si/x4 sequences indeed form the start of the major si/x4 branch in the tree. Mo, months; n.t., not tested.

**Figure 5:**
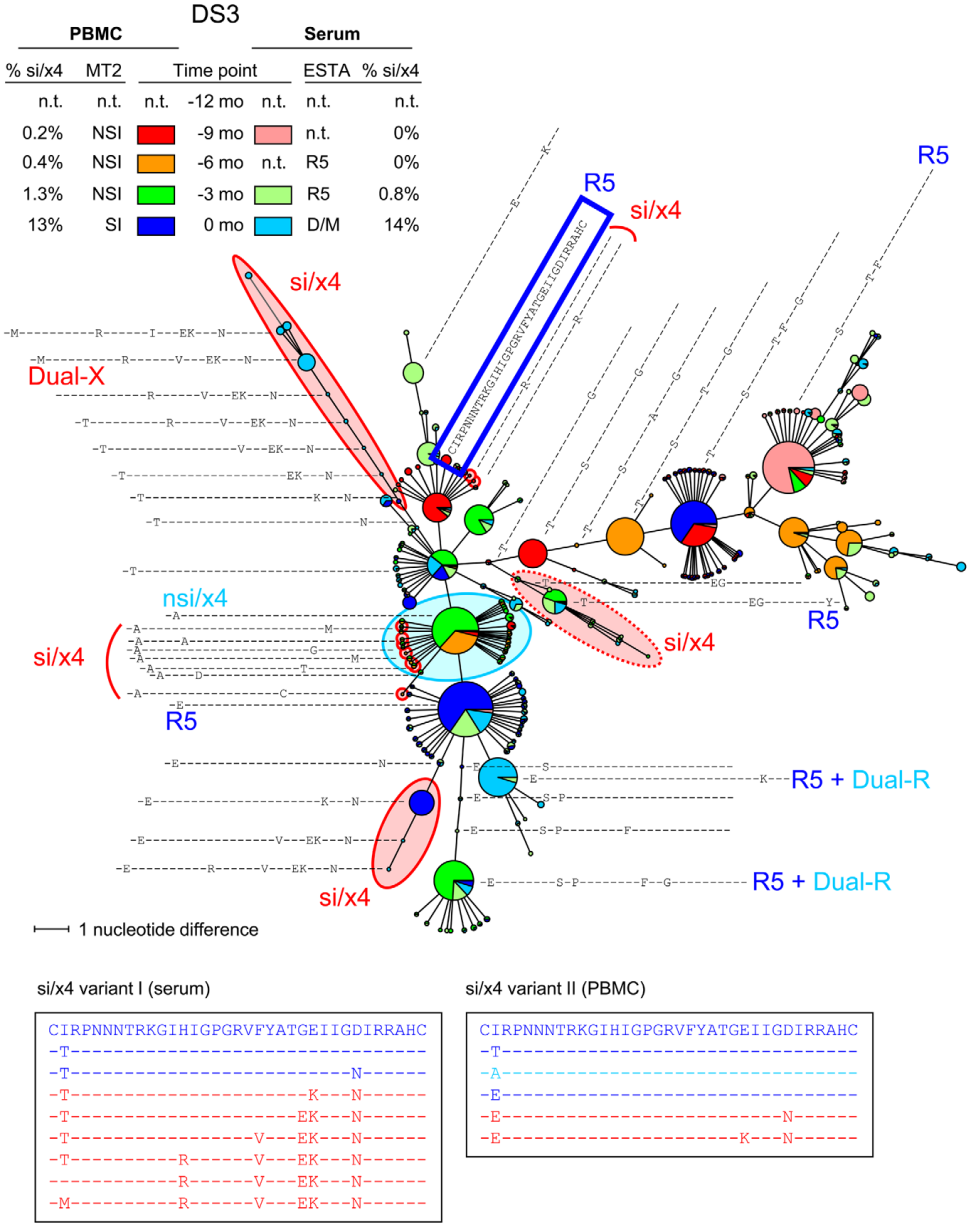
Multiple si/x4 variants emerge in subject DS3. An MST of all V3 sequences generated from PBMC and serum samples of all time points is shown. See the legend to Figure 3 for details regarding the layout of the figure. Nodes containing V3 sequences with an si phenotype as inferred by PSSM_NSI/SI_ and an x4 phenotype as inferred by geno2pheno_[coreceptor]_ are indicated in red, nodes containing discordant nsi/x4 variants are indicated in light blue, while all other sequences were predicted to be nsi/r5. Two distinct si/x4 branches and several minor si/x4 variants can be observed in this MST, which are described in more detail in the text. In addition, one branch contains V3 sequences with a predicted si/x4 phenotype which were reported R5 in the Trofile assay (indicated by dotted red oval). N.t., not tested; mo, months.

Similar to subject DS1, relatively little sequence variation was observed among the nsi/r5 variants in subjects DS4 (**Figure S2**), and DS5 (**Figure S3**). In these individuals, eight or fewer major amino acid sequence variants represented more than 80% of all nsi/r5 reads for every time point and compartment (**Table 4**). Many of these variants were present at multiple time points, and major shifts in variants from one time point to the next were not observed. In contrast, many different major nsi/r5 sequences (11 or more) were present in subjects DS2 (shown as an example in **Figure 4**), DS3 (**Figure 5**), DS6 (**Figure S4**), DS7 (**Figure S5**) and DS8 (**Figure S6**) both at any one time point and over time. For example, the major nsi/r5 variant in PBMC at time point −12 months in subject DS2 was completely replaced by other nsi/r5 variants three months later, some of which in turn did not persist at the next time point. At the later time points, the initial major nsi/r5 variant was observed again, but at a lower frequency, while new sequence variants continued to appear. Interestingly, the appearance of an si/x4 variant in this individual at time point zero was preceded by a variant with a predicted nsi/x4 phenotype. The discrepancy between the two phenotype prediction tools for this variant may indicate that this sequence represents an intermediate step in the pathway from R5-to-X4 evolution, as its score was relatively close to the cutoffs for the PSSM and g2p (i.e. −2.49 and 2.6%, respectively). Alternatively, the phenotype of such intermediate variants may not be predicted correctly as these variants are not often analyzed for coreceptor usage *in vitro* and are therefore most likely not included in the set of training sequences for the bioinformatic algorithms. Intermediate nsi/x4 or si/r5 variants were also observed at relatively low frequencies for subjects DS4 (**Figure S2**), DS5 (**Figure S3**), and DS6 (**Figure S4**), whereas a major nsi/x4 variant was observed in subject DS3 (**Figure 5**), and a major si/r5 variant was present at all time points in subject DS8 (**Figure S6**).

### Number of Different si/x4 Variants

While the MSTs of most individuals show only one si/x4 branch, multiple major si/x4 variants appeared in subjects DS3 and DS6. In DS3, two major si/x4 variants appeared at time point zero, one of which was mainly found in serum, while the other was mainly observed in PBMCs (**Figure 5**). These two variants seemed to be highly related, and their distinct branches indeed clustered in the NJ tree (data not shown). The MST shows a third branch containing sequences with an inferred si/x4 phenotype. However, the phenotype of an Env clone with the major V3 sequence from this branch was R5 in the Trofile assay (**Figure 5**), suggesting that the prediction for sequences in this branch was incorrect. In addition, several minor variants with an si/x4 prediction were observed at time points −9 months, −6 months, and −3 months, none of which made up more than 0.15% of the total number of reads per time point. These variants were not detected at any other time point, suggesting that the fitness of these variants was not sufficient to persist, or that they represent sequences with PCR/sequence errors. In subject DS6, two major si/x4 branches were observed, one of which represented an si/x4 variant that was detected in serum only (**Figure S4**). The second branch contained the major si/x4 variant present in PBMCs at time point zero, which was preceded by variants that again were only found in serum.

In subject DS8, the first major si/x4 variant (frequency ≥10% of all reads per time point) was observed at time point zero (**Figure S6**). However, several minor si/x4 variants were detected up to 12 months earlier (frequencies <1.2%). Some of these minor si/x4 variants were not related to the major si/x4 variant that appeared later in infection, while others contained mutations that were also found in the major si/x4 variant, such as an arginine residue at positions 10 or 13 of the V3 loop. In subject DS2, two minor si/x4 variants were observed in serum at time point −3 months (together comprising 0.11% of the total number of reads from that sample), which were no longer detected at time point zero when a third, apparently more successful si/x4 variant (frequency >2% in PBMCs) emerged (**Figure 4**).

## Discussion

The emergence of detectable CXCR4-using variants during HIV-1 infection is a major determinant for disease progression, but is still poorly understood. In this study, we provide a detailed analysis of the kinetics and mutational pathways involved in the appearance of CXCR4-using variants during natural infection using V3 sequences generated by deep sequencing from PBMC and serum samples of eight HIV-1-infected individuals, in combination with V3-based coreceptor prediction tools, in the year before CXCR4-using variants were for the first time detected in the MT-2 assay.

Our sequence analyses show that the transition in coreceptor usage from CCR5 to CXCR4 follows a stepwise mutational pathway. In most subjects, we were able to detect all transitional intermediate variants. These intermediate variants typically emerged in chronological order, indicating that the mutations were introduced sequentially. Many intermediate variants were present at much lower frequencies than the major nsi/r5 and si/x4 variants, indicative of a reduced replication capacity and consistent with a model in which the transition from CCR5- to CXCR4-usage involves the evolution of HIV-1 through a fitness valley [17]. Alternatively, such variants may preferentially replicate in other compartments than PBMCs or serum, which could also explain their limited detection in our study. In agreement with a study by Shankarappa *et al.* [40], we observed a more rapid outgrowth of si/x4 viruses with a substitution at position 11 of the V3 loop to more than 40% of the total number of reads in PBMC at time point zero, as compared to si/x4 viruses without a substitution at this position, indicating that specific mutations in the V3 loop may affect replication kinetics. Unfortunately, the use of ultra-deep sequencing techniques restricted our analysis to the V3 loop, and prevented us from investigating other changes in the viral envelope that may influence coreceptor usage and viral fitness, in particular substitutions in the V1V2 region that may compensate loss-of-fitness mutations in V3 [18].

The specific mutational pathway that led to CXCR4-usage was different for viruses from each individual, and is likely to be at least partially constrained by the viral background. In subject DS3, we observed the emergence of three different si/x4 variants, two of which were closely related. The third predicted CXCR4-using variant contained a different V3 loop, yet showed a similar evolutionary pathway in which a substitution at position 25 of the V3 loop was followed by the introduction of a glutamic acid at position 24 (**Figure 5**). The same phenomenon was observed in subject DS1, from which we also analyzed PBMC and serum samples obtained three and six months after time point zero (data not shown). At these later time points, we observed the appearance of a second predicted CXCR4-using variant, unrelated to the initial si/x4 variant that emerged nine months earlier, but with identical amino acid substitutions at positions 10 and 11 of the V3 loop (data not shown). These observations support data suggesting that the evolution in coreceptor usage is restricted by a limited number of potential transitional pathways [17]. It will be worthwhile to analyze the PBMC and serum samples from all other subjects obtained three and six months after time point zero to study the subsequent evolution of predicted CXCR4-using viruses that were detected in this study, and to determine whether new, unrelated predicted CXCR4-using variants, as observed in subject DS1, appear in other individuals as well.

Despite constraints on the mutational pathways that lead from CCR5- to CXCR4-usage and the low fitness of transitional intermediate variants, CXCR4-using viruses eventually appear in about 50% of subtype B HIV-1-infected individuals prior to the development of AIDS [5]. The selective mechanisms driving emergence of CXCR4-using variants are still not well understood, and may include the accumulation of random mutations resulting in a CXCR4-using virus with a high replicative fitness, or changes in the host environment, such as immune pressure or the availability of target cell (reviewed by Regoes *et al.* [41]). As we only focused on evolution of the V3 loop during transition from CCR5- to CXCR4-usage, we cannot draw any conclusions about potential other factors involved in this process.

The use of deep sequencing in this study allowed us to detect minority variants that would go unnoticed using conventional sequencing techniques. In three of eight individuals, we observed predicted CXCR4-using variants present at extremely low frequencies. These variants may represent transitions from CCR5- to CXCR4-usage that did not compete with a successfully replicating CXCR4-using variant. However, even though we only analyzed reads with a frequency of 3 or more, we cannot exclude that some of these minority sequence variants may have resulted from errors introduced during the PCR or sequencing procedures. Variation in the major sequences detected among different samples from one subject may to some extent be due to stochastic founder events in the RT-PCR or PCR performed prior to ultra-deep sequencing. We have attempted to minimize stochastic sampling effects by performing the RT-PCR reactions for RNA and the PCR reactions for DNA in triplicate and merging these in equal quantities before ultra-deep sequencing. Sequence variants with a frequency >1% in plasma were in general detected longitudinally, indicative of accurate sampling of the major variants in this compartment, while this was much less the case for PBMC samples.

It is known that low-level CXCR4-using variants may be selected upon CCR5 antagonist treatment [42]–[45], but it remains to be determined whether their presence predicts the outgrowth of a major CXCR4-using variant during natural infection or is of pathological relevance in untreated individuals. A recent study in a small number of HIV-1 individuals in whom low-level predicted CXCR4-using variants were detected early in infection showed that these variants could either persist or disappear over time [46]. To determine the relevance of these minority predicted CXCR4-using variants, it would be worthwhile to additionally analyze whether predicted CXCR4-using variants are also present in HIV-infected individuals in whom phenotypic assays continue to detect only CCR5-using variants. In addition, our results showed that the emergence of predicted CXCR4-using variants was preceded by variants with discordant phenotype predictions (nsi/x4 or si/r5) in a subset of individuals. These viruses may represent intermediate stages in the transition from CCR5- to CXCR4-usage. Analysis of V3 sequences of HIV-1 from individuals in whom phenotypic assays do not detect CXCR4-using viruses could also shed light on the question whether the presence of such virus variants is predictive for the appearance of CXCR4-using virus variants, which would argue for combining the results of both predictors for evaluation of patient samples.

The higher prevalence of CXCR4-using viruses in PBMCs compared to serum as observed here and in previous studies [36], [37] suggests that the PBMC compartment may provide the easiest source for the detection of CXCR4-using variants. However, although CXCR4-using variants may preferentially be present in a cell-associated state, we previously observed a good concordance between phenotypic detection of CXCR4-using variants in the MT-2 assay (using PBMCs) and the enhanced-sensitivity Trofile assay (using serum). We here extend this finding by showing that predicted CXCR4-using viruses are not generally detected earlier in one compartment compared to the other. In three of eight individuals, predicted CXCR4-using variants emerged earlier in serum than in PBMCs, while these viruses were first detected in PBMCs in one additional individual, although a difference of one time point in the moment of detection may also result from stochastic variation introduced during sampling or subsequent experimental procedures. These results indicate that analyzing both sources could contribute to the enhanced accuracy of the detection of CXCR4-using viruses.

The recent availability of CCR5 antagonists as anti-HIV therapeutics has highlighted the need to accurately identify CXCR4-using variants in patient samples when considering use of this new drug class. In this study, we show that coreceptor phenotype prediction using V3 sequences generated by deep sequencing allows a more sensitive detection of CXCR4-using HIV-1 variants present at levels below approximately 2.5% of the total virus population during natural infection as compared to the phenotypic MT-2 assay and ESTA. In individuals treated with maraviroc [47] or vicriviroc [48], minority CXCR4-using variants present at less than 1% of the total pre-treatment HIV population can be subject to positive selection and as a result cause virological failure [45], [49], indicating that this level of sensitivity may be clinically relevant for the detection of minor CXCR4-using virus populations. The use of genotypic methods for the detection of CXCR4-using HIV-1 variants may however be limited by the accuracy of the various bioinformatic tools for predicting the correct coreceptor phenotype. For two of ten individuals initially selected for this study, both PSSM and geno2pheno could not distinguish between viruses with different *in vitro* phenotypes. In subject DS9, determinants for coreceptor usage located outside the V3 region were likely to be involved, as phenotypically distinct clones had identical V3 loop sequences. Although some attempts have been made, too few sequences with coreceptor determinants outside V3 have been characterized to incorporate into a reliable prediction algorithm. Moreover, due to the absence of minority CXCR4-using variants in the training sets for these bioinformatic algorithms, the phenotype prediction for low-level CXCR4-using variants from clinical samples may not always be reliable [34]. Despite these shortcomings, recent data have shown that deep sequencing combined with coreceptor prediction efficiently predicts clinical efficacy of CCR5 antagonist therapy (Swenson *et al.*, CROI 2010). This suggests that relatively few individuals harbor these minority and/or difficult to predict variants for a significant period of time. However, improvement of the currently available coreceptor prediction tools may be necessary.

In conclusion, our results show that HIV-1 evolves from CCR5- to CXCR4-usage by the sequential introduction of mutations in the V3 loop of the viral envelope. The observation that intermediate variants were present at much lower frequencies than the major CCR5- or CXCR4-using variants confirms that this process is highly constrained by sequence and fitness requirements of the virus, and may explain why CXCR4-using variants, unlike CCR5-using variants, are not detected in all patients at every stage of disease. These results provide a better understanding of the emergence of CXCR4-using variants during natural infection and may contribute to a more accurate detection of CXCR4-using viruses in HIV-infected individuals for whom CCR5 antagonist treatment is considered.

## Materials and Methods

### Ethics Statement

The Amsterdam Cohort Studies on HIV-1 infection and AIDS (ACS) have been conducted in accordance with the ethical principles set out in the Declaration of Helsinki, and written informed consent was obtained prior to data and material collection. The study was approved by the Academic Medical Center institutional medical ethics committee.

### Subjects

The individuals included in our present study were men who have sex with men participating in the ACS who were seropositive at enrollment into the cohort between 1988 and 1995. All subjects were infected with subtype B HIV-1 and did not receive anti-retroviral therapy at the time of sampling. In the ACS, cocultures of peripheral blood mononuclear cells (PBMCs) from HIV-1-infected individuals and the MT-2 cell line were routinely performed for each visit at approximately three-months intervals [27]. Ten subjects who reported at least three negative MT-2 scores in the 12 months prior to their first positive MT-2 assay result (time point zero) were initially selected for this study, of whom eight were analyzed in detail (**Tables 2** and **3**). In a previous study, a high degree of concordance between the detection of CXCR4-using virus variants in these individuals by the MT-2 assay (using PBMCs) and the enhanced-sensitivity Trofile assay (ESTA; using serum) was observed [35]. For better readability, subject identifiers were recoded as DS1 (H13912), DS2 (H13988), DS3 (H13845), DS4 (H13951), DS5 (H13993), DS6 (H13885), DS7 (H13907), DS8 (H13908), DS9 (H13904), and DS10 (H13940).

### Samples

For each subject we performed deep sequence analysis on plasma and/or PBMC samples collected every 3 months from 12 months prior to the first MT-2 positive time point (time point zero) up to and including time point zero. For the plasma Env clone genotype and in vitro phenotype analysis, plasma samples collected after time point zero were also included.

### Determination of *In Vitro* Coreceptor Phenotype

In the Trofile assay, a population of full-length subject-derived *env* genes is amplified by reverse transcription-PCR and cloned into an Env expression vector library that is used to generate luciferase-reporter pseudoviruses [29]. These are subsequently used to infect U87 target cells expressing CD4 and either CCR5 or CXCR4 coreceptors in a 96-well plate format. Infection is determined by assaying for luciferase activity in the presence and the absence of CCR5 or CXCR4 antagonists, and viral tropism is reported as R5, X4, or dual/mixed (D/M).

To determine the coreceptor phenotype of individual virus variants present in virus populations of HIV-infected individuals, a cloning step was introduced into the protocol by transforming the Env expression vector library into competent cells. Multiple functional *env* clones were subsequently isolated from randomly picked bacterial colonies and were used to produce clonal luciferase-reporter pseudoviruses. Between 7 and 13 clones per serum sample were then tested in the Trofile assay to determine coreceptor phenotype, which was reported as R5, X4 or Dual-tropic. Dual-tropic viruses were further classified as Dual-R and Dual-X variants. Both variants demonstrated infectivity on both CCR5- and CXCR4-expressing cell lines which suppressed in the presence of a specific antagonist. Dual-R variants however demonstrated CXCR4 infectivity only at the lower end of CXCR4 infectivity spectrum. Prior work has demonstrated that the determinants of coreceptor usage in these viruses are most likely located outside of the V3 region [9]. In addition, full-length gp160 sequences were generated to determine the V3 genotype-based prediction of coreceptor tropism (see details below).

### DNA/RNA Extraction and Deep Sequencing

Deep sequencing was performed with minor adaptations to the protocol as described previously by Swenson *et al.* [50]. HIV RNA was extracted from previously frozen serum samples and HIV DNA was extracted from cryopreserved PBMCs, both using a NucliSENS easyMAG (bioMerieux, Marcy l'Etiole, France). The RNA extracts underwent one-step RT-PCR in triplicate (4 µl extract/reaction), while the DNA extracts underwent triplicate first-round PCR. After the first-round PCR, the region encoding the HIV V3 loop was amplified in a second-round PCR using primers designed with Fusion Primers to fuse to the emulsion PCR beads required by the 454 technique. Also included were 12 unique multiplex “barcode” sequence tags to enable the identification of samples after the sequencing was complete. All primers and thermal cycler protocols are listed in **Protocol S1**.

After PCR amplification, the concentrations of the PCR products were quantified using a Quant-iT Picogreen dsDNA Assay Kit (Invitrogen, Carlsbad, CA) and a DTX 880 Multimode Detector (Beckman Coulter, Brea, CA). Triplicate PCRs were then combined in equal proportions (2×10^12^ DNA amplicons from each triplicate sample), purified with Agencourt Ampure PCR Purification beads (Beckman Coulter), requantified, and diluted to a concentration of 2×10^5^ molecules per ml. PCR amplicons were then combined at a ratio of 0.6 molecules∶1 DNA capture microbead for emulsion PCR. Emulsion PCR was performed, and the DNA and beads were washed, purified and prepared for pyrosequencing according to the manufacturer's instructions. The DNA beads were then added onto the 454 pyrosequencing plate (divided into 4 regions) at a density of 250,000 beads per region, as quantified with a Z1 Coulter Particle Counter (Beckman Coulter). The sequence amplified on each bead was determined by pyrosequencing on the GS-FLX [49], [51].

This process (using the standard amplicon GS-FLX technique) generated ∼250 base pairs of data in each direction per amplicon. A typical V3 loop consisted of 105 base pairs (35 amino acids). Truncated reads (defined as sequences missing ≥4 bases at the 5′ or 3′ end of the V3 loop) were not included in the analysis. To reduce the number of sequences affected by PCR or sequencing errors, reads with a frequency of 1 or 2 were excluded from the dataset. The sequence alignments were subsequently inspected manually, and reads containing ambiguous bases (Ns) or out-of-frame insertions or deletions and reads that did not cover the complete V3 region were removed. For all individuals except DS1, a small number of sequences (on average 131 reads per individual, range 46–387) did not cluster with the remaining sequences from that subject in the neighbor-joining tree and/or minimum spanning tree (see details below). In most patients, several unrelated outliers were observed, both within a sample and across samples from different time points, making it unlikely that these may have been derived from a superinfecting virus variant present at extremely low levels. Moreover, these sequences were in most cases identical to one of the major V3 variants from another subject, and were therefore deleted from the dataset as contaminants.

### Determination of Inferred Coreceptor Usage

HIV coreceptor usage was inferred from V3 genotype of each individual sequence generated from the viral population of a sample. Coreceptor usage inferences were made using the bioinformatic algorithms position-specific scoring matrix (PSSM_NSI/SI_) [33] and geno2pheno_[coreceptor]_ (g2p) [34] scoring. Non-genotypic factors such as CD4^+^ cell count were not included in the bioinformatic analysis. PSSM values below the predetermined cutoff of −1.75 were called nsi, whereas those with scores greater than or equal to −1.75 were called si. The g2p method used a 3.5% false-positive rate, with samples categorized as r5 or x4. These cutoffs were originally optimized and validated to predict virologic outcomes on maraviroc using a separate dataset of patients from three clinical trials of maraviroc in treatment-experienced patients (McGovern *et al.*, European AIDS conference 2009; Swenson *et al.*, IDSA Annual Meeting 2009). The cutoffs can be thought of as more “conservative” than the default cutoffs for the algorithms. Note that the lowercase letters were used for these classifications to indicate that tropism had been inferred from genotypic data. Note also that nsi and si correspond roughly to r5 and x4, respectively. CXCR4-usage was conservatively defined to be present when both algorithms were concordant in si/x4 prediction.

### Phylogenetic Analysis

Unique forward and reverse nucleotide sequences from all time points per subject from both PBMC and serum samples were aligned using ClustalW in the software package of BioEdit [52], and edited manually. The matrix of aligned nucleotide sequences was imported into the tree building software PAUP* [53] (http://paup.csit.fsu.edu/), and a neighbour-joining tree [54] was constructed under the Hasegawa-Kishino-Yano (HKY85) model of evolution [55]. We then used all forward and reverse V3 nucleotide sequences from all time points and compartments per subject to construct minimum spanning trees. V3 sequences were first aligned by the unweighted-pair group method using average linkages (UPGMA) with BioNumerics 6.1 software (Applied Maths). A minimum spanning tree was subsequently constructed using a categorical coefficient. The Priority rules parameters were set at the default values for every analysis, and hypothetical nodes were not allowed. For increased readability, minor variants with a non-si/x4 predicted phenotype present at only one time point and in one compartment, and located in ‘dead-end’ branches were removed from the tree.

### Nucleotide Sequence Accession Numbers

V3 nucleotide sequences obtained by conventional sequencing of Env clones tested in the Trofile assay are available from GenBank (accession numbers JF507726 to JF508136). V3 sequences obtained by ultra-deep sequencing are available upon request.

## Supporting Information

Figure S1 Longitudinal detection of V3 sequences. For V3 sequences that were detected at low (<1%), intermediate (1–10%), or high (≥10%) frequencies at the first of two consecutive time points, the percentage of sequences that is also detected at the next time point is shown. For sequences detected in PBMCs at the first of two time points, the lower light grey part of the bar indicates sequences that were again detected in PBMC at the next time point, while the upper dark grey part of the bar represents sequences that were not detected in PBMCs but were instead found in serum at the next time point. For sequences detected in serum at the first of two time points, the lower dark grey part of the bar indicates sequences that were again detected in serum at the next time point, while the upper light grey part of the bar represents sequences that were not detected in serum but were instead found in PBMCs at the next time point.
(TIF)

Figure S2 MST of V3 sequences of subject DS4. This individual shows a very linear evolution from CCR5- to CXCR4-using variants with a stepwise introduction of mutations towards CXCR4-usage. The earliest si/x4 sequence appears in PBMCs at time point −3, and is replaced by an si/x4 variant with additional mutations at time point zero. Only one si/x4 branch is observed. In addition, relatively little variation occurs among the major nsi/r5 variants over time. Mo, months; n.t., not tested.
(TIF)

Figure S3 MST of V3 sequences of subject DS5. In this individual, a major si/x4 variant is already detected at time point −12 months and remains present at all subsequent time points. Similarly, one major nsi/r5 variant is present at all time points in both PBMCs and serum. Altogether, a relatively small number of major nsi/r5 variants is observed, although some of these are restricted to one time point and/or one compartment. The nsi/r5 and si/x4 branches of the tree are connected by an intermediate variant with a predicted si/r5 phenotype. Mo, months; n.t., not tested.
(TIF)

Figure S4 MST of V3 sequences of subject DS6. This individual shows large variation in the major nsi/r5 variants, with many nsi/r5 variants being present for only a short period of time or only in one compartment. Originating from the same major nsi/r5 variant, two distinct si/x4 branches can be observed, both of which contain an intermediate variant with discordant phenotype prediction (nsi/x4, indicated in light blue). Si/x4 variants are first detected in serum at time point −6 months, and do not appear in PBMCs until time point zero. Mo, months; n.t., not tested.
(TIF)

Figure S5 MST of V3 sequences of subject DS7. The first si/x4 variants in this individual are detected in PBMCs and serum at time point −3 months. These minor variants are subsequently replaced by a major si/x4 variant with additional mutations at time point zero. Only one branch of si/x4 variants is observed in this individual, and no discordant nsi/x4 or si/r5 sequences are found. Mo, months; n.t., not tested.
(TIF)

Figure S6 MST of V3 sequences of subject DS8. In contrast to all other individuals where si/x4 variants are only located in external branches of the tree, the major si/x4 branch in the MST of this subject originates from an internal node. This internal si/x4 variant is already present at time point −6 months. Moreover, several minor si/x4 variants are detected as early as time point −12 months, but most of these do not persist at the later time points. A major variant present at all time points has a predicted si/r5 phenotype. The phenotype of Env clones with this V3 sequence was R5 (*n*  =  2) or Dual-R (*n* = 8) in the Trofile assay. In addition, the phenotype of an Env clone corresponding to the node branching off this major variant at the top left side (with an additional substitution at position 27 of the V3 loop) was Dual-X, while it phenotype was predicted to be si/r5. Mo, months; n.t., not tested.
(TIF)

Table S1 Predicted phenotypes and V3 sequences of longitudinally isolated Env clones of subject DS1 for which coreceptor usage was determined in the Trofile assay.
(PDF)

Table S2 Predicted phenotypes and V3 sequences of longitudinally isolated Env clones of subject DS2 for which coreceptor usage was determined in the Trofile assay.
(PDF)

Table S3 Predicted phenotypes and V3 sequences of longitudinally isolated Env clones of subject DS3 for which coreceptor usage was determined in the Trofile assay.
(PDF)

Table S4 Predicted phenotypes and V3 sequences of longitudinally isolated Env clones of subject DS4 for which coreceptor usage was determined in the Trofile assay.
(PDF)

Table S5 Predicted phenotypes and V3 sequences of longitudinally isolated Env clones of subject DS5 for which coreceptor usage was determined in the Trofile assay.
(PDF)

Table S6 Predicted phenotypes and V3 sequences of longitudinally isolated Env clones of subject DS7 for which coreceptor usage was determined in the Trofile assay.
(PDF)

Table S7 Predicted phenotypes and V3 sequences of longitudinally isolated Env clones of subject DS8 for which coreceptor usage was determined in the Trofile assay.
(PDF)

Table S8 Predicted phenotypes and V3 sequences of longitudinally isolated Env clones of subject DS9 for which coreceptor usage was determined in the Trofile assay.
(PDF)

Table S9 Predicted phenotypes and V3 sequences of longitudinally isolated Env clones of subject DS10 for which coreceptor usage was determined in the Trofile assay.
(PDF)

Protocol S1 Primers and thermal cycler protocols for amplification of the HIV V3 region prior to 454-sequencing.
(PDF)
