# Supplementary figures and images for: Detection of Inferred CCR5- and CXCR4-Using HIV-1 Variants and Evolutionary Intermediates Using Ultra-Deep Pyrosequencing
Source: PLoS Pathog. 2011 Jun 23;7(6):e1002106. doi: 10.1371/journal.ppat.1002106 (PMC3121885; doi:10.1371/journal.ppat.1002106)

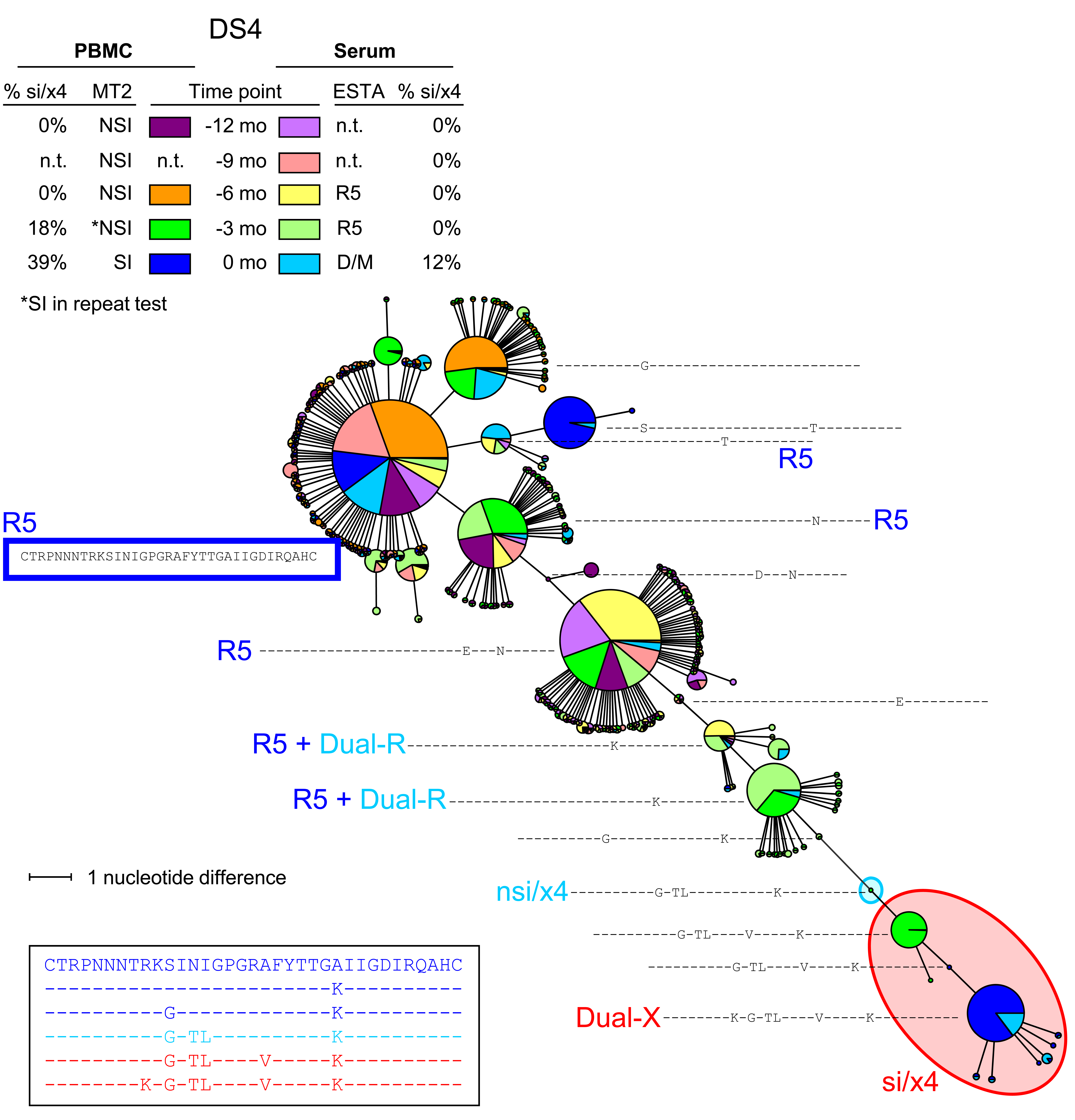

Supplement: Figure S2 — MST of V3 sequences of subject DS4. This individual shows a very linear evolution from CCR5- to CXCR4-using variants with a stepwise introduction of mutations towards CXCR4-usage. The earliest si/x4 sequence appears in PBMCs at time point −3, and is replaced by an si/x4 variant with additional mutations at time point zero. Only one si/x4 branch is observed. In addition, relatively little variation occurs among the major nsi/r5 variants over time. Mo, months; n.t., not tested. (TIF) [file ppat.1002106.s002.tif]
