# Supplementary material for: Detection of Inferred CCR5- and CXCR4-Using HIV-1 Variants and Evolutionary Intermediates Using Ultra-Deep Pyrosequencing
Source: PLoS Pathog. 2011 Jun 23;7(6):e1002106. doi: 10.1371/journal.ppat.1002106 (PMC3121885; doi:10.1371/journal.ppat.1002106)
Supplement: Table S1 — Predicted phenotypes and V3 sequences of longitudinally isolated Env clones of subject DS1 for which coreceptor usage was determined in the Trofile assay. (PDF) [file ppat.1002106.s007.pdf]

Table S1: Predicted phenotypes and V3 sequences of longitudinally isolated Env clones of subject DS1 for which coreceptor usage was determined in the Trofile assay.

Time point (mo to T0)	*n* clones	Phenotype Trofile	Predicted phenotype (PSSM/g2p)	V3 sequence[a] CTRPNNNTRKSISIGPGRAFYATGEIIGDIRQAHC
-6	8	R5	nsi/r5	-----------------------------------
	1	R5	nsi/r5	------------------------Q----------
	1	R5	nsi/r5	-I--------D-H-----------.-V--------
0	4	R5	nsi/r5	-----------------------------------
	1	R5	nsi/r5	------------------------G----------
	4	Dual-X	si/x4	---------QR------------RQ----------
	1	Dual-X	si/x4	---------QR-----------IRQ----------
	1	Dual-X	si/x4	---------ER------------RQ----------

[a] V3 amino acid sequences are shown relative to the major sequence in PBMCs at time point -9 months as determined by ultra-deep sequencing. A dot indicates a deletion in the V3 loop at that position.
