# Supplementary material for: Detection of Inferred CCR5- and CXCR4-Using HIV-1 Variants and Evolutionary Intermediates Using Ultra-Deep Pyrosequencing
Source: PLoS Pathog. 2011 Jun 23;7(6):e1002106. doi: 10.1371/journal.ppat.1002106 (PMC3121885; doi:10.1371/journal.ppat.1002106)
Supplement: Table S2 — Predicted phenotypes and V3 sequences of longitudinally isolated Env clones of subject DS2 for which coreceptor usage was determined in the Trofile assay. (PDF) [file ppat.1002106.s008.pdf]

Table S2: Predicted phenotypes and V3 sequences of longitudinally isolated Env clones of subject DS2 for which coreceptor usage was determined in the Trofile assay.

Time point (mo to T0)	*n* clones	Phenotype Trofile	Predicted phenotype (PSSM/g2p)	V3 sequence[a] CIRPNNNTRKGIHIGPGRAFYTTGEIIGDIRQAHC
-9	4	R5	nsi/r5	-T--------S-P----------------------
	1	R5	nsi/r5	-T--------S-----------------N----S-
	1	R5	nsi/r5	-T--------------------------N----S-
	1	R5	nsi/r5	-A---------------------------------
-6	5	R5	nsi/r5	-T---------------------------------
	4	R5	nsi/r5	-T--------S-P----------------------
	1	R5	nsi/r5	-T--------S-P----K-----------------
	1	R5	nsi/r5	-T--------S------K-----------------
	1	R5	nsi/r5	-T--------S-----------------N----S-
0	9	R5	nsi/r5	-T--------S----------A------N----S-
	1	R5	nsi/r5	-T--------S-P----------------------
	1	R5	nsi/r5	-T--------S------K-----------------
	1	R5	nsi/r5	-T--------S-----------------N----F-
11	5	R5	nsi/r5	-T--------S-P----------------------
	5	R5	nsi/r5	-T--------S------K-----------------
14	3	R5	nsi/r5	-T---------------------------------
	3	R5	nsi/r5	-T--------S-P----------------------
	2	R5	nsi/r5	-T--------S------K-----------------
	2	Dual-X	si/x4	-T----Y-----Y------V----K----------
	1	Dual-X	si/x4	-T----Y-----Y----K-V----K----------

[a] V3 amino acid sequences are shown relative to the major sequence in PBMCs at time point -12 months as determined by ultra-deep sequencing.
