# Supplementary material for: Detection of Inferred CCR5- and CXCR4-Using HIV-1 Variants and Evolutionary Intermediates Using Ultra-Deep Pyrosequencing
Source: PLoS Pathog. 2011 Jun 23;7(6):e1002106. doi: 10.1371/journal.ppat.1002106 (PMC3121885; doi:10.1371/journal.ppat.1002106)
Supplement: Table S3 — Predicted phenotypes and V3 sequences of longitudinally isolated Env clones of subject DS3 for which coreceptor usage was determined in the Trofile assay. (PDF) [file ppat.1002106.s009.pdf]

Table S3: Predicted phenotypes and V3 sequences of longitudinally isolated Env clones of subject DS3 for which coreceptor usage was determined in the Trofile assay.

Time point (mo to T0)	*n* clones	Phenotype Trofile	Predicted phenotype (PSSM/g2p)	V3 sequence[a] CIRPNNNTRKGIHIGPGRVFYATGEIIGDIRRAHC
-6	4	R5	nsi/r5	-E---------------------------------
	2	R5	nsi/r5	-T----------------T-F--------------
	2	R5	nsi/r5	-I--------S-------T-F--------------
	1	R5	nsi/r5	----------S-------T-F----------K---
	1	R5	nsi/r5	-T--------S-------T-F--------------
	4	Dual-R	nsi/r5	-E---------------------------------
	1	Dual-R	si/r5	-E----------------------G----------
-3	2	R5	nsi/r5	-E--------S-P-------F---G----------
	1	R5	nsi/r5	----------G-------T-F------------Y-
	1	R5	nsi/r5	-T--------S-------T-F------------Y-
	1	R5	nsi/r5	-T--------S-P-------F---G----------
	1	Dual-R	nsi/r5	-E--------S-P-------F---G----------
	1	Dual-R	nsi/r5	----------------------------------
	1	Dual-R	nsi/r5	-T------------------F----------K---
	1	Dual-R	nsi/r5	----------S-------T-F----------K---
	1	Dual-R	nsi/r5	-------------------------------K---
0	3	R5	nsi/r5	-E--------S-P-------F---G----------
	1	R5	nsi/r5	-E---------------------------------
	1	R5	nsi/r5	-E-----------------------------K---
	1	Dual-R	nsi/r5	-E---------------------------------
	1	Dual-R	nsi/r5	-E--------S-P-------F---G----------
	1	Dual-R	nsi/r5	-E-----------------------------K---
	2	Dual-X	si/x4	-M----------R------V---EK---N------
6	2	R5	nsi/r5	-E------------------F----V-------Y-
	2	R5	nsi/r5	-E----------------T-F------------Y-
	1	R5	si/x4	-T---------------------EG--------Y-
	1	R5	nsi/r5	-E---------------------------------
	3	Dual-R	nsi/r5	-E---------------------------------
	2	Dual-R	nsi/r5	-E------------------F----V-------Y-
	2	Dual-X	si/x4	-M----------Y------V---EK---N------
	1	Dual-X	si/x4	-M----------S------V---EK---N------
9	1	R5	nsi/r5	-E----------------T-F------------Y-
	1	Dual-R	nsi/r5	-T------------------F----V-------Y-
	1	Dual-R	nsi/r5	-E------------------F----V-------Y-
	1	Dual-R	nsi/r5	-T------------------F------------Y-
	4	Dual-X	si/x4	-E----------A------V---EK---N------
	2	Dual-X	si/x4	-M----------A------V---EK---N------
	1	Dual-X	si/x4	-E----------A------V---EK---N------
	1	Dual-X	si/x4	-M----------S------V---EK---N------
	1	Dual-X	si/x4	-M----------S------V---EK----------
	1	Dual-X	si/x4	-E----------S------V---EK---N------

[a] V3 amino acid sequences are shown relative to the major sequence in PBMCs at time point -9 months as determined by ultra-deep sequencing.
