# Supplementary material for: Detection of Inferred CCR5- and CXCR4-Using HIV-1 Variants and Evolutionary Intermediates Using Ultra-Deep Pyrosequencing
Source: PLoS Pathog. 2011 Jun 23;7(6):e1002106. doi: 10.1371/journal.ppat.1002106 (PMC3121885; doi:10.1371/journal.ppat.1002106)
Supplement: Table S4 — Predicted phenotypes and V3 sequences of longitudinally isolated Env clones of subject DS4 for which coreceptor usage was determined in the Trofile assay. (PDF) [file ppat.1002106.s010.pdf]

Table S4: Predicted phenotypes and V3 sequences of longitudinally isolated Env clones of subject DS4 for which coreceptor usage was determined in the Trofile assay.

Time point (mo to T0)	*n* clones	Phenotype Trofile	Predicted phenotype (PSSM/g2p)	V3 sequence[a] CTRPNNNTRKSINIGPGRAFYTTGAIIGDIRQAHC
-6	4	R5	nsi/r5	-----------------------------------
	2	R5	nsi/r5	-------I---------------------------
	1	R5	nsi/r5	----------------------------N------
	1	R5	nsi/r5	------------------------E---N------
-3	3	R5	nsi/r5	------------------------K----------
	6	Dual-R	nsi/r5	------------------------K----------
0	8	R5	nsi/r5	------------------------T----------
	4	Dual-X	si/x4	-------IK-G-TL----V-----K----------
6	4	R5	nsi/r5	-----T------------------E---N------
	2	R5	nsi/r5	-------R---------------------------
	1	R5	nsi/r5	------------------------E---N------
	5	Dual-X	si/x4	--------K-G-TL----V-----K-V--------
9	6	R5	nsi/r5	------------------------E---N------
	2	R5	nsi/r5	-------R---------------------------
	1	R5	nsi/r5	------------------------E---N-K----
	1	R5	nsi/r5	------------------------T----------
	2	Dual-X	si/x4	--------K-G-TL----V-----K-V--------
	1	Dual-X	si/x4	--------K-G-TL----V-----K----------

[a] V3 amino acid sequences are shown relative to the major sequence in PBMCs at time point -12 months as determined by ultra-deep sequencing.
