# Supplementary material for: Detection of Inferred CCR5- and CXCR4-Using HIV-1 Variants and Evolutionary Intermediates Using Ultra-Deep Pyrosequencing
Source: PLoS Pathog. 2011 Jun 23;7(6):e1002106. doi: 10.1371/journal.ppat.1002106 (PMC3121885; doi:10.1371/journal.ppat.1002106)
Supplement: Table S5 — Predicted phenotypes and V3 sequences of longitudinally isolated Env clones of subject DS5 for which coreceptor usage was determined in the Trofile assay. (PDF) [file ppat.1002106.s011.pdf]

Table S5: Predicted phenotypes and V3 sequences of longitudinally isolated Env clones of subject DS5 for which coreceptor usage was determined in the Trofile assay.

Time point (mo to T0)	*n* clones	Phenotype Trofile	Predicted phenotype (PSSM/g2p)	V3 sequence[a] CTRPNNNTRKGIHIGPGRTFYATGEIIGDIRQAHC
-6	9	R5	nsi/r5	-----------------------------------
-3	7	R5	nsi/r5	----------S------------------------
	2	R5	nsi/r5	-----------------------------------
	1	R5	nsi/r5	--------------------HV-------------
0	7	R5	nsi/r5	-----------------------------------
	2	R5	nsi/r5	----------S------------------------
	1	R5	nsi/r5	--G-------S------------------------
	1	R5	nsi/r5	---------RS-------ALFTA-----N------
3	5	R5	nsi/r5	----------S------------------------
	1	R5	nsi/r5	-----------------------------M-----
	1	R5	nsi/r5	---------RS-------A-FTA-----N------
	2	Dual-X	si/x4	---------RS-Y-----ARFTA-K------K-Y-
6	3	R5	nsi/r5	-----------------------------------
	1	R5	nsi/r5	--------------------------V--------
	1	R5	nsi/r5	---------RS-Y----------------------
	7	Dual-X	si/x4	---------RS-Y-----ARFTA-K------K-Y-

[a] V3 amino acid sequences are shown relative to the major sequence in PBMCs at time point -12 months as determined by ultra-deep sequencing.
