# Supplementary material for: Detection of Inferred CCR5- and CXCR4-Using HIV-1 Variants and Evolutionary Intermediates Using Ultra-Deep Pyrosequencing
Source: PLoS Pathog. 2011 Jun 23;7(6):e1002106. doi: 10.1371/journal.ppat.1002106 (PMC3121885; doi:10.1371/journal.ppat.1002106)
Supplement: Table S6 — Predicted phenotypes and V3 sequences of longitudinally isolated Env clones of subject DS7 for which coreceptor usage was determined in the Trofile assay. (PDF) [file ppat.1002106.s012.pdf]

Table S6: Predicted phenotypes and V3 sequences of longitudinally isolated Env clones of subject DS7 for which coreceptor usage was determined in the Trofile assay.

Time point (mo to T0)	*n* clones	Phenotype Trofile	Predicted phenotype (PSSM/g2p)	V3 sequence[a] CTRPNNNTRRSINIGPGRAFYTTGQIIGDIRQAHC
-6	7	R5	nsi/r5	----G-----------------.-E----------
	2	R5	nsi/r5	---------K-------------------------
	1	R5	nsi/r5	----G-----------------.-E-V--------
	1	R5	nsi/r5	----G-----------------.-EM---------
	1	R5	nsi/r5	----G---G-------------.-E----------
0	3	R5	nsi/r5	----G-----------------.-E---N------
	2	R5	nsi/r5	----G-----------------.-E----------
	1	R5	nsi/r5	----G-------------------D---N------
	1	R5	nsi/r5	----G-----------------.-E---NL-----
	3	Dual-X	si/x4	---------KR-SL----VY-----------K---
	1	Dual-X	si/x4	---------KR-SL----VY---------MRK---

[a] V3 amino acid sequences are shown relative to the major sequence in PBMCs at time point -12 months as determined by ultra-deep sequencing. A dot indicates a deletion in the V3 loop at that position.
