# Supplementary material for: Detection of Inferred CCR5- and CXCR4-Using HIV-1 Variants and Evolutionary Intermediates Using Ultra-Deep Pyrosequencing
Source: PLoS Pathog. 2011 Jun 23;7(6):e1002106. doi: 10.1371/journal.ppat.1002106 (PMC3121885; doi:10.1371/journal.ppat.1002106)
Supplement: Table S7 — Predicted phenotypes and V3 sequences of longitudinally isolated Env clones of subject DS8 for which coreceptor usage was determined in the Trofile assay. (PDF) [file ppat.1002106.s013.pdf]

Table S7: Predicted phenotypes and V3 sequences of longitudinally isolated Env clones of subject DS8 for which coreceptor usage was determined in the Trofile assay.

Time point (mo to T0)	*n* clones	Phenotype Trofile	Predicted phenotype (PSSM/g2p)	V3 sequence[a] CTRPSNNTRQGIHIGPGRAFYATTKIIGDIRQAYC
-7	4	R5	nsi/r5	-------------------I-.--D------K---
	2	R5	nsi/r5	-----------------------------------
	2	Dual-R	nsi/r5	-----------------------------------
	2	Dual-R	si/r5	-------------------------------K---
	2	Dual-R	si/x4	---------R--Y------------------K---
-3	3	R5	nsi/r5	---------KS--------L--.-D------K---
	1	R5	nsi/r5	-----------------------------------
	1	R5	nsi/r5	-------------------I-.--N------K---
	1	R5	nsi/r5	----N---KKSVN------I-.--D------K---
	1	R5	si/r5	-------------------------------K---
	4	Dual-R	si/r5	-------------------------------K---
	1	Dual-X	si/r5	--------------------------V----K---
	1	Dual-X	si/x4	---------R---V-----I-----------K---
	1	Dual-X	si/x4	---------R---------------------K---
0	2	R5	nsi/r5	----------SV-------L-.--N------K---
	1	R5	nsi/r5	----N-----SV-------L-.--N------K---
	1	R5	nsi/r5	----N-----SV-------L-.--D------K---
	1	R5	nsi/r5	-------------------I-.--N------K---
	1	R5	si/r5	-------------------------------K---
	1	R5	si/r5	----------SV-------L-T---------K---
	2	Dual-R	si/r5	-------------------------------K---
	2	Dual-X	si/x4	---------R---------I-----------K---
	1	Dual-X	si/x4	---------R--R------I-----------K---

[a] V3 amino acid sequences are shown relative to the major sequence in PBMCs at time point -12 months as determined by ultra-deep sequencing. A dot indicates a deletion in the V3 loop at that position.
