# Supplementary material for: Detection of Inferred CCR5- and CXCR4-Using HIV-1 Variants and Evolutionary Intermediates Using Ultra-Deep Pyrosequencing
Source: PLoS Pathog. 2011 Jun 23;7(6):e1002106. doi: 10.1371/journal.ppat.1002106 (PMC3121885; doi:10.1371/journal.ppat.1002106)
Supplement: Table S8 — Predicted phenotypes and V3 sequences of longitudinally isolated Env clones of subject DS9 for which coreceptor usage was determined in the Trofile assay. (PDF) [file ppat.1002106.s014.pdf]

Table S8: Predicted phenotypes and V3 sequences of longitudinally isolated Env clones of subject DS9 for which coreceptor usage was determined in the Trofile assay.

Time point (mo to T0)	*n* clones	Phenotype Trofile	Predicted phenotype (PSSM/g2p)	V3 sequence[a] CVRPGNNTRKSITIGPGKAFYT-REIIGNIRQAHC
-3	8	R5	nsi/x4	-M-------------------A-G----D--K---
	3	R5	nsi/x4	-M---------------R---A-G----D--K---
	1	R5	nsi/x4	-M---------------R---A-G-----------
	1	dual-R	nsi/x4	-M-------------------A-G----D--K---
0	1	R5	nsi/r5	-M------K------------A-G----D--K---
	6	R5	nsi/x4	-M-------------------A-G----D--K---
	1	R5	nsi/x4	-M---------------R---A-G----D--K---
	1	R5	nsi/x4	-M-------R-------R---A-G----D--K---
	1	R5	nsi/x4	-M----------A----R---A-G----D--K---
	1	dual-X	nsi/x4	-M---------------R---A-G----D--K---
1	5	R5	nsi/x4	-M-------------------A-G----D--K---
	2	R5	nsi/x4	-M-------R-------R---A-G----D--K---
	2	R5	nsi/x4	-M---------------R---A-G----D--K---
	1	dual-X	nsi/x4	-M---------------R---A-G----D--K---
	1	dual-X	nsi/x4	-M-------------------A-G----D--K---
3	1	R5	nsi/r5	---------R-------R---A-G----D--K---
	4	R5	nsi/x4	-M-------------------A-G----D--K---
	2	R5	nsi/x4	-M---------------R---A-G----D--K---
	1	R5	nsi/x4	-M-------R-------R---A-G----D--K---
	1	dual-X	nsi/x4	-M---------------R---A-G----D--K---
	1	dual-X	nsi/x4	-M-------------------A-G----D--K---
4	1	R5	nsi/r5	---------R-------R---A-G----D--K---
	5	R5	nsi/x4	-M-------------------A-G----D--K---
	2	R5	nsi/x4	-M-------R-------R---A-G----D--K---
	2	R5	nsi/x4	-M---------------R---A-G----D--K---
	5	dual-X	nsi/x4	-M---------------R---A-G----D--K---

[a] V3 amino acid sequences are shown relative to the major sequence in PBMCs at time point -12 months as determined by ultra-deep sequencing.
