# Supplementary material for: Detection of Inferred CCR5- and CXCR4-Using HIV-1 Variants and Evolutionary Intermediates Using Ultra-Deep Pyrosequencing
Source: PLoS Pathog. 2011 Jun 23;7(6):e1002106. doi: 10.1371/journal.ppat.1002106 (PMC3121885; doi:10.1371/journal.ppat.1002106)
Supplement: Table S9 — Predicted phenotypes and V3 sequences of longitudinally isolated Env clones of subject DS10 for which coreceptor usage was determined in the Trofile assay. (PDF) [file ppat.1002106.s015.pdf]

Table S9: Predicted phenotypes and V3 sequences of longitudinally isolated Env clones of subject DS10 for which coreceptor usage was determined in the Trofile assay.

Time point (mo to T0)	*n* clones	Phenotype Trofile	Predicted phenotype (PSSM/g2p)	V3 sequence[a] CTRPNNNTKRDIHIGPGR-AFYATE-IVGDIRQAHC
-3	1	R5	si/r5	-----------P----------D----------
	1	R5	si/x4	--------AKG---------M----R---N--R---
	7	Dual-R	si/x4	--------A-G---------M---QR------R---
	1	Dual-R	si/x4	--------AKG---------M---QR------R---
0	4	R5	nsi/r5	--------R-N---------M---GQ-I-N------
	1	R5	nsi/r5	--------RK----------M---GQ-I-N------
	1	R5	si/r5	--------RK--------------D-----------
	3	R5	si/x4	--------A-G---------M---QR------R---
	1	R5	si/x4	--------AKG---------M---QR------R---
	1	R5	si/x4	--------AKG---------M----R------R---
	1	R5	si/x4	--------AKG--L------M---QR------R---
	1	Dual-R	si/x4	--------A-G---------M---QR------R---
3	1	R5	nsi/r5	--------RK----------M---GE------R---
	1	R5	si/r5	-----------P----------D----------
	3	R5	si/x4	--------A-G---------M---QR------R---
	7	Dual-R	si/x4	--------A-G---------M---QR------R---
	1	Dual-R	si/r5	--------R-G-------AI-F---R---N------
6	6	R5	si/r5	-----------P----------D----------
	1	R5	si/x4	--------AKG---------M---QR------R---
	1	Dual-R	si/x4	--------AKG---------M---QR------R---
	5	Dual-X	si/x4	--------A-G-R------VI--AKR------R---

[a] V3 amino acid sequences are shown relative to the major sequence in PBMCs at time point -12 months as determined by ultra-deep sequencing.
