# Supplementary material for: Detection of Inferred CCR5- and CXCR4-Using HIV-1 Variants and Evolutionary Intermediates Using Ultra-Deep Pyrosequencing
Source: PLoS Pathog. 2011 Jun 23;7(6):e1002106. doi: 10.1371/journal.ppat.1002106 (PMC3121885; doi:10.1371/journal.ppat.1002106)
Supplement: Protocol S1 — Primers and thermal cycler protocols for amplification of the HIV V3 region prior to 454-sequencing. (PDF) [file ppat.1002106.s016.pdf]

Primers for 454 genotyping

First-round PCR primer, forward: 5’ GAGCCAATTCCCATACATTATTGT 3’
First-round PCR primer, reverse: 5’ GCCCATAGTGCTTCCTGCTGCTCCCAAGAACC 3’

Second-round PCR primer, forward: 5’ AATGCCAAAACCATAATAGTACA 3’
Second-round PCR primer, reverse: 5’ GAAAAATTCCCTTCCACAATTAAA 3’

Fusion primer: 5’ GCCTCCCTCGCGCCATCAG 3’

Deep sequencing barcode tags:

(**A**) ACGAGTGCGT
(**B**) ACGCTCGACA
(**C**) AGACGCACTC
(**D**) AGCACTGTAG
(**E**) ATCAGACACG
(**F**) CGTGTCTCTA
(**G**) CTCGCGTGTC
(**H**) TAGTATCAGC
(**I**) TCTCTATGCG
(**J**) TGATACGTCT
(**K**) TACTGAGCTA
(**L**) ATATCGCGAG

Example of complete deep sequencing forward primer, tag A:

(fusion primer, then *barcode A*, then **PCR primer**)

5’ GCCTCCCTCGCGCCATCAG*ACGAGTGCGT***AATGCCAAAACCATAATAGTACA** 3’

Thermal cycler protocols

RT-PCR

30’	52°C	
2’	94°C	
15’’	94°C	40x
30’’	55°C	
1’30’’	68°C	
5’	68°C	

Second-round PCR

2’	94°C	
15’’	94°C	35x
30’’	55°C	
50’’	72°C	
5’	72°C	
